# Vital Signs: Disparities in Nonsmokers’ Exposure to Secondhand Smoke — United States, 1999–2012

**Published:** 2015-02-06

**Authors:** David M. Homa, Linda J. Neff, Brian A. King, Ralph S. Caraballo, Rebecca E. Bunnell, Stephen D. Babb, Bridgette E. Garrett, Connie S. Sosnoff, Lanqing Wang

**Affiliations:** 1 Office on Smoking and Health, National Center for Chronic Disease Prevention and Health Promotion, CDC; 2 Division of Laboratory Sciences, National Center for Environmental Health, CDC

## Abstract

**Background:**

Exposure to secondhand smoke (SHS) from burning tobacco causes disease and death in nonsmoking children and adults. No risk-free level of SHS exposure exists.

**Methods:**

National Health and Nutrition Examination Survey (NHANES) data from 1999–2012 were used to examine SHS exposure among the nonsmoking population aged ≥3 years. SHS exposure among nonsmokers was defined as a serum cotinine level (a metabolite of nicotine) of 0.05–10 ng/mL. SHS exposure was assessed overall and by age, sex, race/ethnicity, poverty level, education, and whether the respondent owned or rented their housing.

**Results:**

Prevalence of SHS exposure in nonsmokers declined from 52.5% during 1999–2000 to 25.3% during 2011–2012. During this period, declines were observed for all population subgroups, but disparities exist. During 2011–2012, SHS was highest among: children aged 3–11 years (40.6%), non-Hispanic blacks (46.8%), persons living below the poverty level (43.2%), and persons living in rental housing (36.8%). Among children aged 3–11 years, 67.9% of non-Hispanic blacks were exposed to SHS compared with 37.2% of non-Hispanic whites and 29.9% of Mexican Americans.

**Conclusion:**

Overall, SHS exposure in the United States has been reduced by half since 1999–2000. However, 58 million persons were still exposed to SHS during 2011–2012, and exposure remains higher among children, non-Hispanic blacks, those living in poverty, and those who rent their housing.

**Implications for Public Health Practice:**

Eliminating smoking in indoor spaces fully protects nonsmokers from SHS exposure; separating smokers from nonsmokers, cleaning the air and ventilating buildings cannot completely eliminate exposure. Continued efforts to promote implementation of comprehensive statewide laws prohibiting smoking in workplaces and public places, smoke-free policies in multiunit housing, and voluntary smoke-free home and vehicle rules are critical to protect nonsmokers from this preventable health hazard in the places they live, work, and gather.

## Introduction

Exposure to secondhand smoke (SHS) from burning tobacco products causes sudden infant death syndrome (SIDS), respiratory infections, ear infections, and asthma attacks in infants and children, and coronary heart disease, stroke, and lung cancer in adult nonsmokers (1,2). No risk-free level of SHS exposure exists (2). SHS exposure causes more than 41,000 deaths among nonsmoking adults and 400 deaths in infants each year, and approximately $5.6 billion annually in lost productivity (1,3). Although population exposure to SHS has declined over the past 2 decades (3,4), many nonsmokers remain exposed to SHS in workplaces, public places, homes, and vehicles (5).

## Methods

Data from the 1999–2012 National Health and Nutrition Examination Survey (NHANES) were analyzed to assess the most recent trends and correlates of SHS exposure among nonsmokers aged ≥3 years. NHANES is a complex, multistage survey representative of the noninstitutionalized U.S. population. Since 1999, NHANES has been conducted in continuous 2-year cycles. NHANES includes a home interview, physical examination at a mobile examination center where biologic specimens are collected, and laboratory specimen testing, including serum cotinine analysis, for participants aged ≥3 years.* Interview response rates ranged from 72.6% (2011–2012) to 84.0% (2001–2002); examination response rates ranged from 69.5% (2011–2012) to 80.0% (2001–2002).†

SHS exposure was assessed using serum cotinine, a metabolite of nicotine that reflects recent exposure (4,6). Serum cotinine values are based on analysis of blood samples collected by venipuncture from consenting participants; laboratory analysis is performed using an isotope dilution liquid chromatography tandem mass spectrometry method (4). The limit of detection for serum cotinine initially was 0.05 ng/mL but changed to 0.015 ng/mL because of improvements in the method (4). Serum cotinine concentrations >10 ng/mL are associated with recent active smoking (6). Therefore, children aged 3–11 years were considered nonsmokers if their cotinine concentration was ≤10 ng/mL. Adolescents aged 12–19 years were considered nonsmokers if their cotinine concentration was ≤10 ng/mL and they did not report smoking within the preceding 30 days or using any nicotine-containing product within the preceding 5 days. Adults aged ≥20 years were considered nonsmokers if their cotinine concentration was ≤10 ng/mL and they did not report being a current smoker§ or use of any nicotine-containing product within the preceding 5 days. The numbers of nonsmokers with serum cotinine data in each survey cycle ranged from 5,742 to 6,540.

For each survey cycle, the percentage of nonsmokers aged ≥3 years with serum cotinine levels of 0.05–10 ng/mL, an established standard for classifying SHS exposure (the lower cutpoint of 0.05 ng/mL allows for historical comparisons) (3), was computed overall and by sex, age, race/ethnicity,¶ poverty status, and education; housing status (own or rent) was also assessed as a proxy for multiunit housing residency.** Wald 95% confidence limits were computed for all percentages, and differences were assessed using a two-sided Student’s t-test (p<0.05). Data are presented for 1999–2000, 2003–2004, 2007–2008, and 2011–2012.†† For 2011–2012, the most recent NHANES cycle, the estimated number of nonsmokers with serum cotinine levels 0.05–10 ng/mL was calculated by race/ethnicity and age group using midpoint population estimates from the 2011–2012 American Community Survey.§§ Examination weights were used in analysis to account for the complex sample design and differential probability of sample selection, nonresponse, and noncoverage.

## Results

The proportion of U.S. nonsmokers aged ≥3 years with serum cotinine levels 0.05–10 ng/mL declined from 52.5% during 1999–2000 to 25.3% during 2011–2012 (percentage change = 51.8%) (Table 1). By age, declines were least among children aged 3–11 years (percentage change = 37.4%) and greatest among adults aged ≥20 (percentage change = 55.6%). By race/ethnicity, declines in SHS exposure were least among non-Hispanic blacks (percentage change = 36.6%), followed by Mexican Americans (percentage change = 46.0%) and non-Hispanic whites (percentage change = 56.2%). By poverty level, declines in exposure were less among those living below the poverty level (percentage change = 39.7%) than those living at or above this level (percentage change = 56.6%). By education, lesser declines in SHS exposure were generally observed among those with lower levels of educational attainment. By housing status, a lesser decline in exposure was observed among those who rented their housing (percentage change = 46.0%) than those who owned their housing (percentage change = 58.5%).

During 2011–2012, prevalence of SHS exposure was higher among children aged 3–11 years (40.6%) and adolescents aged 12–19 years (33.8%) than adults aged ≥20 years (21.3%). By race/ethnicity, prevalence was higher among non-Hispanic blacks (46.8%) than Mexican Americans (23.9%) and non-Hispanic whites (21.8%). Prevalence was higher among persons living below the poverty level (43.2%) than persons living at or above the poverty level (21.2%). By education, prevalence was highest among persons with grade 11 or less education (27.6%) and lowest among persons with a college diploma or greater education (11.8%). By housing status, prevalence was higher among persons who rented their housing (36.8%) than persons who owned their housing (19.0%).

Among children aged 3–11 years, prevalence of SHS exposure declined comparably from 1999–2000 to 2011–2012 among non-Hispanic whites (percentage change = 41.2%) and Mexican Americans (percentage change = 39.0%); however, a lesser decline was observed among non-Hispanic blacks (percentage change = 19.8%) (Figure). During 2011–2012, SHS exposure among children aged 3–11 years was significantly higher among non-Hispanic blacks (67.9%) than non-Hispanic whites (37.2%; p<0.05) and Mexican Americans (29.9%; p<0.05) (Table 2). Among adolescents aged 12–19 years and adults aged ≥20 years, prevalence was significantly higher among non-Hispanic blacks (54.6% and 39.6%) than non-Hispanic whites (35.8% and 17.9%; p<0.05) and Mexican Americans (16.9% and 23.8%; p<0.05).

During 2011–2012, an estimated 57.9 million nonsmokers aged ≥3 years were exposed to SHS (Table 3). Of these, approximately 15.1 million were aged 3–11 years, 9.6 million were aged 12–19 years, and 35.2 million were aged ≥20 years. By race/ethnicity, 31.3 million non-Hispanic white nonsmokers aged ≥3 years were exposed, including 7.2 million children aged 3–11 years; 12.4 million non-Hispanic black nonsmokers aged ≥3 years were exposed, including 3.4 million children aged 3–11 years; and 6.2 million Mexican American nonsmokers aged ≥3 years were exposed, including 1.9 million children aged 3–11 years.

## Conclusions and Comment

From 1999–2000 to 2011–2012, SHS exposure among U.S. nonsmokers declined overall and among all population groups. However, during 2011–2012, an estimated one quarter of U.S. nonsmokers, or 58 million persons, were still exposed to SHS, including 15 million children aged 3–11 years. Moreover, declines in exposure over time have been slower, and prevalence of exposure remains higher, among children, non-Hispanic blacks, persons living in poverty, and persons who rent their housing. The Surgeon General has concluded that eliminating smoking in indoor spaces fully protects nonsmokers from SHS exposure (2). Continued efforts to promote comprehensive statewide laws prohibiting smoking in workplaces and public places, voluntary smoke-free rules prohibiting smoking in homes and vehicles at all times, and smoke-free policies in multiunit housing are critical to protect nonsmokers from this preventable health hazard in the places they live, work, and gather (2,7,8).

Several factors might have contributed to the declines in SHS exposure. First, over the past 25 years, almost 700 local municipalities have implemented comprehensive smoke-free laws that prohibit smoking in indoor areas of worksites, restaurants, and bars (9); additionally, 26 states and the District of Columbia have implemented such laws since 2002 (10). Almost half (49.3%) of U.S. residents are currently covered by comprehensive smoke-free laws at the state or local level.¶¶ Second, increasing numbers of households have adopted voluntary smoke-free home rules; the proportion of U.S. households with smoke-free rules increased from 43.0% during 1992–1993 to 83.0% during 2010–2011 (11). Third, substantial changes have occurred in social norms regarding the acceptability of smoking around nonsmokers (2). Finally, cigarette smoking prevalence has declined (1,12).

Despite this progress, millions of U.S. nonsmokers remain exposed to SHS, and disparities in exposure exist. During 2011–2012, prevalence was higher among children aged 3–11 years (40.6%) than all other age groups. This finding might reflect the recent slowing in the decline in adult smoking prevalence and the persistence of smoking in homes (11,12). The home is the primary source of exposure for children (2), and nearly all nonsmokers who live with someone who smokes inside their home are exposed to SHS (5). Exposure was also higher among non-Hispanic blacks, including nearly seven in 10 children. Non-Hispanic black nonsmokers historically have higher cotinine levels than nonsmokers of other race/ethnicities (2,4,13). The reasons for this difference are uncertain, but biologic evidence suggests that slower metabolism of cotinine might result in blacks having higher cotinine levels for a given level of exposure (14). Other possible reasons relate to racial/ethnic variations in smoke-free policy coverage in workplaces and public settings (15), as well as smoke-free rules in homes and vehicles (16); for example, among employed U.S. adults, workplace SHS exposure among non-Hispanic blacks (25.6%) was higher than that of their white counterparts (17.7%) (15). Similarly, among all U.S. adults, SHS exposure was higher among non-Hispanics blacks than whites in homes (11.4% versus 5.3%) and vehicles (13.6% versus 8.2%) (16). In U.S. households that included both children and smokers during 2006–2007, only 32.8% of non-Hispanic black households had complete home smoking restrictions, compared with 48.0% of non-Hispanic white households and 72.2% of Mexican American households (17). These findings underscore the importance of continued efforts to reduce SHS exposure in all settings to protect nonsmokers, particularly children. Based on evidence that SHS exposure is reduced among children whose parents have been informed about the harms of SHS, the American Academy of Pediatrics and the U.S. Public Health Service recommend that clinicians ask parents about their smoking, advise them about the harms of SHS, and offer encouragement and help quitting (18,19).

Greater SHS exposure was observed among those who rent their housing, a proxy for multiunit housing residency and among those living below the poverty level. Disparities in smoking persist among smokers with low socioeconomic status, which might have contributed to these disparities in SHS exposure (20). Many persons with low socioeconomic status also live in multiunit housing, where SHS can infiltrate smoke-free living units from units and shared areas where smoking occurs; approximately 80 million U.S. residents live in multiunit housing, one quarter of whom live below the poverty level (21). The potential for SHS exposure in subsidized housing is particularly concerning because a large proportion of these units are occupied by persons who are especially sensitive to the effects of SHS, including children, the elderly, and the disabled (22). Prohibiting smoking in all U.S. subsidized housing, including public housing, would generate annual societal cost savings of approximately $500 million (22). The U.S. Department of Housing and Urban Development has encouraged public housing authorities and operators of multifamily housing rental assistance programs (e.g., Section 8), to implement smoke-free policies.*** As of October 2014, several hundred housing authorities had instituted such policies, including all 20 in Maine.††† Continued efforts to implement smoke-free policies in both subsidized and market-rate multiunit housing could further protect nonsmokers from SHS exposure in their homes.

***Key Points***

- There is no safe level of exposure to secondhand smoke (SHS). Eliminating smoking in indoor spaces fully protects nonsmokers from exposure to SHS; separating smokers from nonsmokers, cleaning the air, and ventilating buildings cannot completely eliminate exposure.
- From 1999–2000 to 2011–2012, SHS exposure among U.S. nonsmokers declined overall (from 52.5% to 25.3%) and among all population groups.
- During 2011–2012, one quarter of U.S. nonsmokers, or 58 million persons, were still exposed to SHS, including 15 million children ages 3–11 years.
- Declines in exposure over time have been smaller, and prevalence of exposure remains higher among children, non-Hispanic blacks, persons living in poverty, and persons who rent their housing.
- Continued efforts to promote comprehensive statewide laws prohibiting smoking in workplaces and public places, smoke-free policies in multiunit housing, and voluntary smoke-free home and vehicle rules are critical to protect nonsmokers from this preventable health hazard in the places they live, work, and gather.
- Additional information is available at http://www.cdc.gov/vitalsigns.

The findings in this report are subject to at least five limitations. First, smoking status was based on self-report and serum cotinine levels. Some smokers misrepresent their smoking status in surveys (23); using serum cotinine levels to verify self-reported nonsmoking status should reduce this bias (5). Still, serum cotinine cutpoints can vary by race/ethnicity, age, and background SHS levels (5,13). However, the cutpoint (>10 ng/mL) used to define smokers is widely accepted (5). Second, the NHANES sample design prevented examination of trends among certain other racial/ethnic populations, such as Hispanic subgroups other than Mexican Americans, Asian-Pacific Islanders, American Indian/Alaska Natives, and lesbian/gay/bisexual/transgender persons. Third, NHANES did not directly measure multiunit housing status across all survey cycles; however, a secondary analysis demonstrated strong correlation between rental/own status and multiunit housing residency. Fourth, the prevalence estimates presented are likely conservative, because 0.05 ng/mL is used as the cutpoint defining exposure versus the current limit of detection of 0.015 ng/mL. Finally, nonresponse bias cannot be ruled out because interview response rates ranged from 72.6% to 84.0% and examination response rate ranged from 69.5% to 80.0%.

Although substantial progress has been made in reducing the prevalence of SHS exposure in the United States, disparities persist; 15 million children aged 3–11 years, including seven in 10 non-Hispanic black children, remain exposed to this preventable health hazard. Continued efforts are critical to further reduce SHS exposure, especially among vulnerable populations. Implementation of both comprehensive smoke-free laws in indoor public places and worksites and smoke-free policies in multiunit housing, together with continued adoption of voluntary smoke-free home and vehicle rules, can further reduce nonsmokers’ exposure to SHS (1,2,7,8). Furthermore, continued education regarding the harms of SHS exposure, such as CDC’s “Tips” campaign, can reinforce the benefits of smoke-free environments.§§§

## Figures and Tables

**FIGURE:**
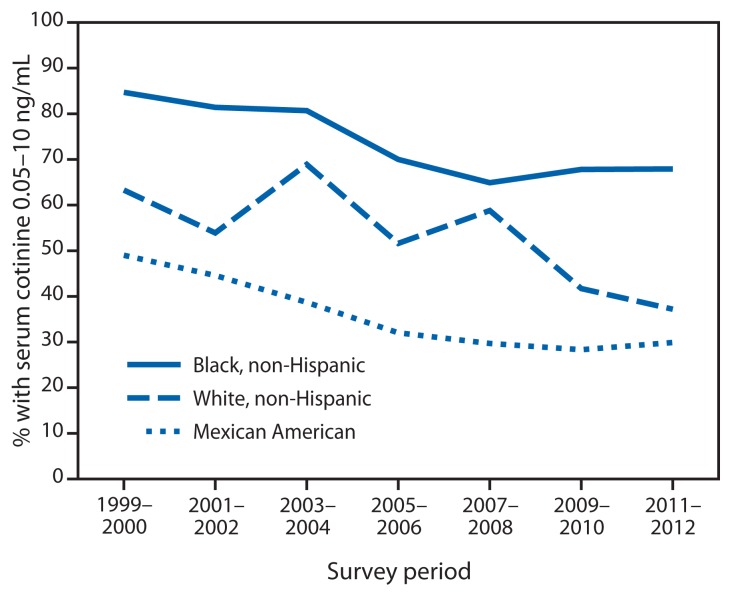
Percentage of nonsmoking children aged 3–11 years with serum cotinine levels 0.05–10 ng/mL, by race/ethnicity^*^ — National Health and Nutrition Examination Survey, United States, 1999–2012 ^*^ Because of the sample design, analyses of data by race/ethnicity were limited to the three racial/ethnic populations available across all survey cycles (non-Hispanic whites, non-Hispanic blacks, and Mexican Americans).

**TABLE 1:** Percentage of nonsmokers with serum cotinine levels 0.05–10 ng/mL, by selected demographic characteristics — National Health and Nutrition Examination Survey, United States, 1999–2012

Characteristic	1999–2000		2003–2004		2007–2008		2011–2012		Relative % decline* (1999–2000 to 2011–2012)
			
	%	(95% CI)	%	(95% CI)	%	(95% CI)	%	(95% CI)	
**Total**	**52.5**	**(47.1–57.9)**	**47.6**	**(40.4–54.9)**	**40.1**	**(35.0–45.3)**	**25.3**	**(22.5–28.1)**	**51.8**
**Sex**									
Male	58.5	(52.1–64.9)	51.9	(44.3–59.5)	43.5	(37.5–49.4)	27.7	(24.7–30.6)	52.6
Female	47.5	(42.5–52.5)	44.2	(36.8–51.6)	37.4	(32.6–42.2)	23.3	(20.4–26.3)	50.9
**Age group (yrs)**									
3–11	64.9	(56.0–73.9)	64.8	(55.5–74.2)	53.6	(46.2–61.0)	40.6	(34.0–47.2)	37.4
12–19	63.1	(56.4–69.7)	57.1	(50.3–63.9)	46.5	(38.3–54.8)	33.8	(28.2–39.4)	46.4
≥20	48.0	(42.6–53.4)	42.4	(35.1–49.8)	36.7	(32.0–41.3)	21.3	(18.6–24.0)	55.6
**Race/Ethnicity** †									
White, non-Hispanic	49.8	(42.9–56.7)	46.0	(36.8–55.3)	40.1	(32.2–48.0)	21.8	(18.6–24.9)	56.2
Black, non-Hispanic	73.8	(69.6–77.9)	68.0	(60.0–75.9)	55.9	(50.6–61.3)	46.8	(38.0–55.6)	36.6
Mexican American	44.3	(37.4–51.1)	34.0	(25.5–42.5)	28.5	(23.1–33.9)	23.9	(16.3–31.4)	46.0
**Poverty status**									
<Poverty level	71.6	(64.8–78.5)	63.6	(55.0–72.2)	60.5	(55.0–66.0)	43.2	(37.3–49.0)	39.7
≥Poverty level	48.8	(42.8–54.8)	44.8	(37.7–52.0)	36.9	(31.3–42.5)	21.2	(18.8–23.6)	56.6
Unspecified	53.5	(48.4–58.6)	50.5	(36.4–64.6)	39.6	(30.8–48.5)	31.7	(22.8–40.5)	40.7
**Education (age ≥25 yrs)**									
≤Grade 11	53.9	(48.7–59.0)	48.8	(42.9–54.8)	45.1	(39.3–50.9)	27.6	(23.0–32.2)	48.8
High school diploma or equivalent	51.6	(44.5–58.6)	50.1	(39.8–60.4)	41.4	(33.2–49.7)	27.5	(21.2–33.7)	46.7
Some college or associate degree	48.2	(40.8–55.6)	42.7	(32.1–53.4)	37.6	(30.9–44.2)	21.2	(17.5–24.9)	56.0
≥College diploma	35.2	(27.5–43.0)	29.8	(23.2–36.3)	22.0	(17.2–26.7)	11.8	(9.1–14.4)	66.5
**Own or rent home**									
Own	45.8	(39.3–52.3)	43.5	(35.4–51.6)	35.5	(29.4–41.6)	19.0	(16.1–22.0)	58.5
Rent	68.1	(61.6–74.6)	57.4	(50.8–64.0)	52.7	(48.7–56.7)	36.8	(32.3–41.3)	46.0

**Abbreviation:** CI = confidence interval.

* All declines statistically significant at p<0.05.

† Because of the sample design, analyses of data by race/ethnicity were limited to the three racial/ethnic populations available across all survey cycles (non-Hispanic whites, non-Hispanic blacks, and Mexican Americans).

**TABLE 2:** Percentage of nonsmokers with serum cotinine levels 0.05–10 ng/mL, by age group and race/ethnicity* — National Health and Nutrition Examination Survey, United States, 1999–2012

Characteristic	1999–2000		2003–2004		2007–2008		2011–2012		Relative % decline† (1999–2000 to 2011–2012)
			
	%	(95% CI)	%	(95% CI)	%	(95% CI)	%	(95% CI)	
**Aged 3–11 yrs**									
White, non-Hispanic	63.3	(48.7–78.0)	68.9	(56.8–81.0)	58.8	(47.9–69.6)	37.2	(30.0–44.4)	41.2
Black, non-Hispanic	84.7	(79.2–90.3)	80.7	(70.2–91.2)	64.9	(53.0–76.7)	67.9	(57.1–78.6)	19.8
Mexican American	49.0	(39.1–58.9)	38.7	(28.9–48.6)	29.7	(20.2–39.1)	29.9	(20.4–39.4)	39.0
**Aged 12–19 yrs**									
White, non-Hispanic	61.8	(52.6–71.1)	56.9	(48.0–65.8)	47.9	(33.9–61.8)	35.8	(28.6–43.0)	42.1
Black, non-Hispanic	80.4	(76.0–84.7)	74.0	(67.7–80.4)	60.2	(51.6–68.8)	54.6	(43.0–66.2)	32.1
Mexican American	48.3	(40.8–55.8)	35.1	(26.6–43.6)	29.1	(18.3–39.9)	16.9	(7.0–26.9)	65.0
**Aged ≥20 yrs**									
White, non-Hispanic	45.7	(39.3–52.0)	40.7	(31.6–49.8)	36.3	(29.3–43.3)	17.9	(13.8–21.9)	60.8
Black, non-Hispanic	68.2	(62.5–73.8)	61.7	(52.9–70.5)	52.2	(46.6–57.9)	39.6	(32.6–46.6)	41.9
Mexican American	41.2	(34.0–48.4)	31.9	(22.6–41.1)	28.0	(23.2–32.7)	23.8	(16.2–31.4)	42.2

**Abbreviation:** CI = confidence interval.

* Because of the sample design, analyses of data by race/ethnicity were limited to the three racial/ethnic populations available across all survey cycles (non-Hispanic whites, non-Hispanic blacks, and Mexican Americans).

† All declines statistically significant at p<0.05.

**TABLE 3:** Estimated number of nonsmokers aged ≥3 years with serum cotinine levels 0.05–10 ng/mL, by race/ethnicity* and age group — National Health and Nutrition Examination Survey, United States, 2011–2012

Characteristic	No. of nonsmokers (millions)†	% with serum cotinine 0.05–10 ng/mL	No. with serum cotinine 0.05–10 ng/mL (millions)†	95% CI
**Overall**	**228.8**	**25.3**	**57.9**	**51.5–64.3**
3–19 yrs	64.9	37.3	24.2	20.7–27.7
3–11 yrs	37.1	40.6	15.1	12.6–17.5
12–19 yrs	28.4	33.8	9.6	8.0–11.2
≥20 yrs	165.3	21.3	35.2	30.8–39.7
20–39 yrs	56.3	27.9	15.7	12.8–18.5
40–59 yrs	60.1	19.3	11.6	10.0–13.2
≥60 yrs	49.1	16.2	7.9	5.9–10.0
**White, non-Hispanic**				
≥3 yrs	143.6	21.8	31.3	26.7–35.8
3–19 yrs	34.1	36.5	12.5	10.4–14.4
3–11 yrs	19.5	37.2	7.2	5.8–8.6
12–19 yrs	15.1	35.8	5.4	4.3–6.5
≥20 yrs	110.2	17.9	19.7	15.2–24.1
20–39 yrs	31.6	24.6	7.8	6.0–9.5
40–59 yrs	39.5	16.3	6.4	4.6–8.3
≥60 yrs	39.2	14.0	5.5	3.3–7.6
**Black, non-Hispanic**				
≥3 yrs	26.4	46.8	12.4	10.0–14.7
3–19 yrs	9.2	61.2	5.6	4.6–6.6
3–11 yrs	5.1	67.9	3.4	2.9–4.0
12–19 yrs	4.3	54.6	2.3	1.8–2.8
≥20 yrs	17.3	39.6	6.9	5.6–8.1
20–39 yrs	6.6	50.7	3.3	2.8–3.9
40–59 yrs	6.8	32.3	2.2	1.7–2.7
≥60 yrs	4.0	32.9	1.3	1.0–1.6
**Mexican American**				
≥3 yrs	25.9	23.9	6.2	4.2–8.1
3–19 yrs	10.6	24.0	2.5	1.6–3.5
3–11 yrs	6.3	29.9	1.9	1.3–2.5
12–19 yrs	4.4	16.9	0.7	0.3–1.2
≥20 yrs	15.4	23.8	3.7	2.5–4.8
20–39 yrs	8.3	24.2	2.0	1.1–3.0
40–59 yrs	5.3	24.9	1.3	1.0–1.7
≥60 yrs	1.8	16.6	0.3	0.2–0.4

**Abbreviation:** CI = confidence interval.

* Because of sample size design, analyses of data by race/ethnicity are limited to non-Hispanic whites, non-Hispanic blacks, and Mexican Americans; therefore, race/ethnicity totals do not add up to overall totals.

† Totals do not sum exactly because of rounding.

## Footnotes

* Available at http://www.cdc.gov/nchs/data/series/sr_01/sr01_056.pdf.

† Available at http://www.cdc.gov/nchs/nhanes/response_rates_cps.htm.

§ Adults aged ≥20 years were considered to be self-reported smokers if they reported smoking ≥100 cigarettes in their lifetime and that they were smoking every day or some days at the time of interview.

¶ Because of the NHANES sample design, race/ethnicity analyses were limited to the three racial/ethnic populations available across all survey cycles: non-Hispanic whites, non-Hispanic blacks, and Mexican Americans. However, all race/ethnicity groups are included in the reported values for the total population, as well as the values presented by sex, age group, poverty level, education, and housing status (own or rent).

** From 1999–2000 to 2005–2006, NHANES included a variable describing whether a housing unit was attached (single-family house attached to one or more houses, apartment, or dormitory) or detached (mobile home or trailer, or single-family house detached from any other house). This variable was examined against own/rent status for 1999–2006, with the findings indicating that 65%–68% of renters lived in multiunit housing (defined as an attached single-family home, apartment, or dormitory).

†† The 1999–2000 data cycle was chosen as the baseline data point for presentation because it precedes the period for when statewide comprehensive smoke-free laws were in effect. There were no statewide comprehensive smoke-free laws before 2002. Every other data cycle after 1999–2000 is presented (2003–2004, 2007–2008, and 2011–2012).

§§ Available at http://www.cdc.gov/nchs/data/nhanes/response_rates_cps/acs_totals_1112.pdf.

¶¶ Available at http://no-smoke.org/pdf/SummaryUSPopList.pdf.

*** Available at http://portal.hud.gov/hudportal/documents/huddoc?id=12-25pihn.pdf and http://www.tcsg.org/sfelp/HUD-SFHsgImplemt091510.pdf.

††† Available at http://www.no-smoke.org/pdf/smokefreemuh.pdf.

§§§ Available at http://www.cdc.gov/tobacco/campaign/tips/stories/nathan.html.
